# Successful Treatment of Methimazole-Induced Severe Aplastic Anemia by Granulocyte Colony-Stimulating Factor, Methylprednisolone, and Cyclosporin

**DOI:** 10.5402/2011/732623

**Published:** 2011-04-10

**Authors:** Munehiro Honda

**Affiliations:** ^1^Fourth Department of Medicine, Teikyo University School of Medicine, Kawasaki, Japan; ^2^Department of Endocrinology and Metabolism, Mishuku Hospital, 5-33-12 Kamimeguro, Meguro-ku, Tokyo 153-0051, Japan

## Abstract

A 52-year-old Japanese woman was examined because of general malaise, weight loss and a lump in her left breast. She was diagnosed with cancer of the left breast and Graves' disease, and was administered methimazole (MMI). A left mastectomy was performed for the breast cancer. She presented with a high fever and peripheral blood examination revealed a severe pancytopenia. She was diagnosed with severe aplastic anemia, and administered G-CSF, however, the treatment was unsuccessful. Thus, oral methyprednisolone and cyclosporin were added. There was a remarkable improvement in the peripheral blood count.

## 1. Introduction

Methimazole (MMI) is widely used to treat Graves' disease. Agranulocytosis is a severe side effect and its frequency is reported to be 0.18–0.55% [1–5]. Aplastic anemia (AA) is very rare in MMI treatment and most cases rapidly recover after cessation of MMI therapy [6–13]. Granulocyte colony-stimulating factor (G-CSF), glucocorticoid and cyclosporin have only been used in severe cases. I report here a case of MMI-induced severe AA associated with severe bacterial infection. The patient was treated with G-CSF after cessation of MMI, but her peripheral blood count did not recover even when she was administered a higher dosage of G-CSF for 11 days. Therefore, we administered a combination of methyprednisolone and cyclosporin and, her peripheral blood count and bone marrow rapidly recovered. 

## 2. Case Report

**Figure 1:**
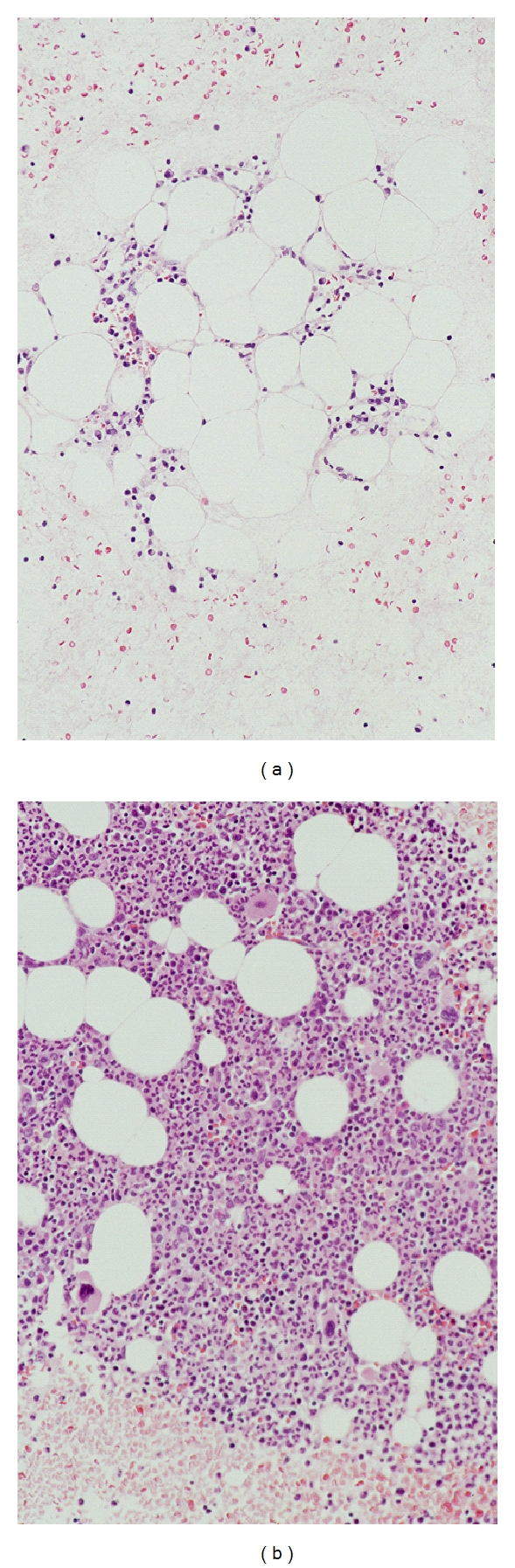
Photomicrographs of bone marrow aspiration. (a) Decreased nucleated cells with fat replacement in bone marrow at diagnosis (b) Increased nucleated cells with an absence of fat replacement (HE staining ×100).

**Figure 2:**
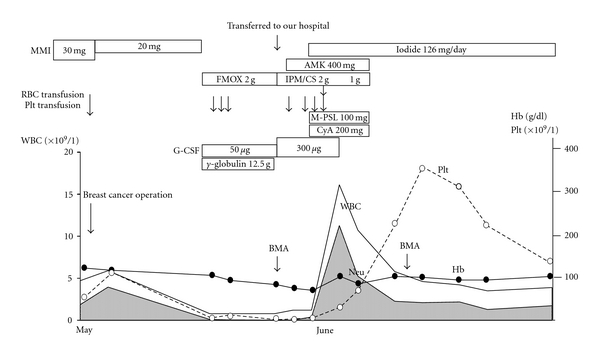
Illustration of the clinical course of the case. MMI: methimazole, FMOX: flomoxef, AMK: amikacin, IPM/CS: imipenem/cilastatin, M-PSL: methyprednisolone, CyA: cyclosporin, BMA: bone marrow aspiration, Neu: neutrophils.

 A 52-year-old Japanese woman was examined in March, 2003 because of general malaise, weight loss and a lump in her left breast. She was diagnosed with cancer of the left breast and Graves' disease, and was administered MMI 30 mg/day for Graves' disease. Her thyroid function was controlled with MMI, and a left mastectomy was performed for breast cancer one month later. She presented with a sudden fever of 39°C and a sore throat 12 days after the operation. The peripheral blood examination revealed a white blood-cell count of 0.8×10^9^/L, with 12% neutrophils, 3% monocytes, 2% eosinophils, 83% lymphocytes, hemoglobin concentration of 9.5 g/dL, and platelet count of 4×10^9^/L; therefore, MMI therapy was stopped and she was administered 50 *μ*g of G-CSF subcutaneous injection and 2.5 g of gamma globulin intravenous infusion. The treatment was unsuccessful, and she was transferred to our hospital because of progressive pancytopenia and a constant high fever for a week. On admission, she complained of a sore throat, chills and general malaise. A physical examination revealed a high fever (39°C), sinus tachycardia, grade II systolic murmur, exophthalmos, moderate diffuse goiter and gingival bleeding. The palpebral conjunctiva was anemic. No superficial lymph nodes were swollen. A Penrose drain was left in her left breast, and exudates were noted. Oropharyngeal examination revealed tonsillitis without white curd-like plaque. Electrocardiogram showed sinus tachycardia. Chest X-ray was normal. Peripheral blood examination showed a white blood-cell count of 0.8×10^9^/L, with 2% neutrophils, hemoglobin concentration of 8.5 g/dL, platelet count of 3×10^9^/L, and reticulation of red blood cells of 1.3%. Laboratory examinations showed normal liver function and renal function. Serum albumin was 3.0 g/dL, alkaline phosphatase was 260 IU/L, total cholesterol was 92 mg/dL, and C-reactive protein (CRP) was 16.9 mg/dL. Throat and blood bacterial cultures were negative, but exudate culture from her left breast yielded Staphylococcus species. Thyroid function tests were TSH <0.002 mIU/L, free triiodothyronine (FT3) >20.0 pg/mL, free thyroxine (FT4) >8.0 ng/dL, and TSH receptor antibody 19.7 IU/L; thus MMI-induced severe AA was strongly suspected, and bone marrow (BM) aspiration was performed immediately. There were decreased nucleated cells with fat replacement in BM (Figure 1(a)). The patient was administered iodide 126 mg/day and imipenem/cilastatin 2 g/day and G-CSF was increased to 300 *μ*g/day. Two days later, intravenous amikacin (400 mg/day) was added; however, with a progressive depression in peripheral blood count, the patient needed transfusions of erythrocytes and platelets. Five days later, oral methyprednisolone and cyclosporin were added. There was a remarkable improvement of the high fever and pancytopenia, and normalization of the CRP. G-CSF was stopped 8 days later, and methyprednisolone and cyclosporin were stopped 10 days later. A second bone marrow aspiration was performed 3 weeks later and BM was already normocellular (Figure 1(b)); thus she was administered ^131^I radioniodine, and 2 months later her thyroid function was normal. The clinical course of this case is illustrated in Figure 2. 

## 3. Discussion

 AA is very rare complication as a result of the administration of MMI [6–13]. The pathogenic mechanisms of agranulocytosis and AA as a result of the administration of MMI are unclear; however, direct cytotoxic effect by MMI or autoimmune reaction for bone marrow precursors are candidates of pathogenic mechanisms. Previously, it was reported that agranulocytosis was developed in patients administered more than 40 mg/day MMI; however, agranulocytosis developed regardless of the dosage and duration of MMI administration [1, 2]. Tamai et al. found that HLA DRB1∗08032 related to MMI induced agranulocytosis in Japanese [14]; however, our patient did not have it. Weitzman and Stossel reported expression of antineutrophil antibody belonging to IgM in the sera of patients who developed MMI induced agranulocytosis [15]. It is reported that incubation with autologous sera of a patient with MMI induced AA collected on earlier days resulted in a reduced number of colony-forming units in culture in peripheral blood mononuclear cells, and autoimmune humoral reactions may occur against myeloid precursors [7]. In addition, MMI induces other autoimmune disorders such as hemolytic anemia, insulin autoimmune syndrome and lupus-like syndrome [16–18]. Thus, the intervention of autoimmune abnormality may be more causal than the cytotoxic effect.

 Most cases of MMI induced AA usually develop within 3 months after the start of therapy [6]. In our patient, bone marrow aplasia occurred about 1.5 months after the start of MMI. Moreover, recovery is usually prompt within 2–5 weeks from the withdrawal of MMI and the start of G-CSF, and bone marrow transplantation and antithymocyte globulin are seldom needed [6]. However, the withdrawal of MMI and the administration of G-CSF for 11 days had little effect on the peripheral blood count in the patient. Therefore, oral methylprednisolone and cyclosporin were added, the neutrophils count rapidly increased and the erythrocyte and platelet counts gradually increased. Escobar-Morreale et al. reported that in the case of MMI induced AA patient who had no response with granulocyte-monocyte colony-stimulating factor for a week, standard immunosuppressive drugs should be susbstituted [8]. As the course of MMI induced AA is highly variable, it is not known whether bone marrow aplasia recovered spontaneously or whether recovery was a result of the administration of G-CSF or methylprednisolone and cyclosporin. It was reported that the recovery of MMI induced agranulocytosis was shortened significantly by the administration of G-CSF and glucocorticoid [19]. However, the administration of G-CSF for 11 days had little effect on the peripheral blood count in our patient. It could not be determined which drug was most effective, but these combinations seemed useful in our patient.

 As the patient developed AA with breast cancer two weeks postoperatively, the Penrose drain was left in her left breast, and exudates were noted. Staphylococcus species was detected from the culture of exudates. Immunosuppressive agents such as glucocorticoid and cyclosporin generally aggravate an infectious disease, resulting in restorative delay. For example, our patient suffered from severe bacterial infection; however, bone marrow rapidly recovered after the immunosuppressive agent combination was administered and the severe infection was resolved immediately after administration of antibiotics. This suggests that bone marrow is rapidly recovered by a combination of G-CSF and an immunosuppressive agent under the appropriate administration of antibiotics in a severe AA patient with severe infection.

In summary, I report the successful treatment of MMI-induced severe AA by G-CSF, methyprednisolone, and cyclosporin. Although AA is a very rare but severe complication of Basedow's disease with MMI treatment, early recovery can be expected as a result of the administration of a combination of G-CSF and immunosuppressive therapy.
